# Blended Ensemble Learning Prediction Model for Strengthening Diagnosis and Treatment of Chronic Diabetes Disease

**DOI:** 10.1155/2022/4451792

**Published:** 2022-07-14

**Authors:** T. R. Mahesh, Dhilip Kumar, V. Vinoth Kumar, Junaid Asghar, Banchigize Mekcha Bazezew, Rajesh Natarajan, V. Vivek

**Affiliations:** ^1^Department of Computer Science and Engineering, Faculty of Engineering and Technology, JAIN (Deemed-to-be University), Bangalore, India; ^2^Department of Computer Science and Engineering, Vel Tech Rangarajan Dr. Sagunthala R&D Institute of Science and Technology, Chennai, India; ^3^Faculty of Pharmacy, Gomal University, Dera Ismail Khan 29050, Khyber Pakhtunkhwa, Pakistan; ^4^Department of Electrical and Computer Engineering, Wollo University, Ethiopia; ^5^Department of Information Technology, University of Technology and Applied Science, Shinas. Sultanate of Oman, Oman

## Abstract

Diabetes mellitus (DM), commonly known as diabetes, is a collection of metabolic illnesses characterized by persistently high blood sugar levels. The signs of elevated blood sugar include increased hunger, frequent urination, and increased thirst. If DM is not treated properly, it may lead to several complications. Diabetes is caused by either insufficient insulin production by the pancreas or an insufficient insulin response by the body's cells. Every year, 1.6 million individuals die from this disease. The objective of this research work is to use relevant features to construct a blended ensemble learning (EL)-based forecasting system and find the optimal classifier for comparing clinical outputs. The EL based on Bayesian networks and radial basis functions has been proposed in this article. The performances of five machine learning (ML) techniques, namely, logistic regression (LR), decision tree (DT) classifier, support vector machine (SVM), *K*-nearest neighbors (KNN), and random forest (RF), are compared with the proposed EL technique. Experiments reveal that the EL method performs better than the existing ML approaches in predicting diabetic illness, with the remarkable accuracy of 97.11%. The proposed ensemble learning could be useful in assisting specialists in accurately diagnosing diabetes and assisting patients in receiving the appropriate therapy.

## 1. Introduction

Diabetes mellitus (DM) is a collection of metabolic disorders indicated by abnormal insulin production as well as its action. This insulin deficiency leads to elevated blood levels of glucose and decreased carbohydrate and protein metabolism [1]. DM is a prevalent endocrine disease that affects roughly 200 million people across the world. Diabetes affected 30.3 million persons in the United States in 2015, accounting for 9.4% of the population. More than one-fourth of them were unaware that they had this condition. One out of four people over the age of 65 has diabetes [2]. Gradually, the human living environment changes dramatically due to increased demand on the health of human beings, human lifestyle, changes in climate, and other environmental changes. Infections and diseases cause millions of individuals to fall unwell and face other harmful health issues, and these problems are quickly increasing every day.

**Figure 1:**
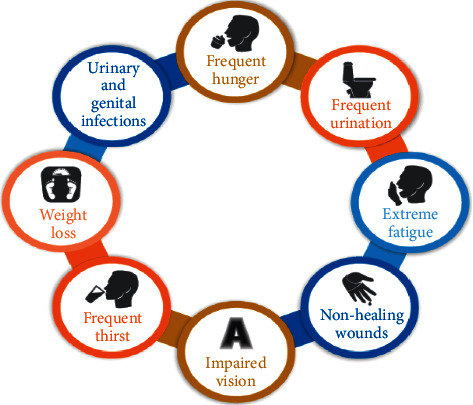
Diabetes' symptoms.

DM is a chronic condition that happens when the pancreas stops producing insulin or when the body's insulin is not used properly. Insulin is a hormone produced by the pancreas that functions as a key to allowing glucose from our diet to move from the bloodstream into our cells for energy production. In the blood, all carbohydrate foods are broken down into glucose. Insulin aids glucose absorption into cells. When the body is unable to make or utilize insulin properly, blood glucose levels rise (known as hyperglycaemia). High glucose levels are linked to long-term harm to the body and the failure of many organs and tissues. Diabetes that is not well controlled can have catastrophic repercussions, including damage to a variety of organs and tissues in the body, including the heart, kidneys, eyes, and nerves. The different symptoms of the DM include frequent hunger, frequent thirst, frequent urination, urinary and genital infections, extreme tiredness, wounds that will not heal, and unexplained weight loss especially in case of children. Because many people do not experience any of these symptoms, diabetes is frequently diagnosed by chance. Those who are at risk of acquiring diabetes should have regular medical examinations to avoid late discovery of the disease. The different symptoms of diabetes are shown in Figure 1.

There are three main kinds of diabetes. Due to the complexity of diabetes, diagnosing and distinguishing among these different kinds are difficult [3–5].

### 1.1. Type-1

An autoimmune reaction occurs when the body's defense system assaults the cells that make insulin, resulting in type-1 diabetes. As a result, the body produces very little insulin. The specific causes are unknown although they are thought to be connected to a combination of genetic and environmental factors. Type-1 diabetes can strike anyone at any age although it strikes children and adolescents the most commonly. When a person has type-1 diabetes, his or her body generates very little or no insulin, necessitating daily insulin injections to keep blood glucose levels in check.

### 1.2. Type-2

**Figure 2:**
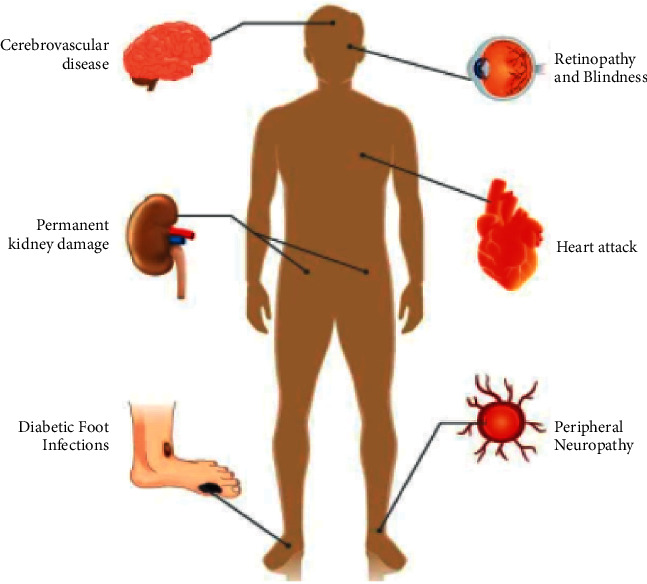
Type-2 diabetes complications.

Type-2 diabetes is the most common form of diabetes, accounting for approximately 90% of all cases. Insulin resistance is a condition in which the body's response to insulin is inadequate. Because insulin is unable to function effectively, blood glucose levels rise, causing more insulin to be released. This can gradually fatigue the pancreas, resulting in the body producing less and less insulin, further raising blood sugar levels (hyperglycaemia). Type-2 diabetes is most typically diagnosed in older persons although it is becoming more common in children, adolescents, and younger adults as obesity, physical inactivity, and poor diet become more prevalent. The major complications that might be caused by type-2 diabetes are depicted in Figure 2.

**Figure 3:**
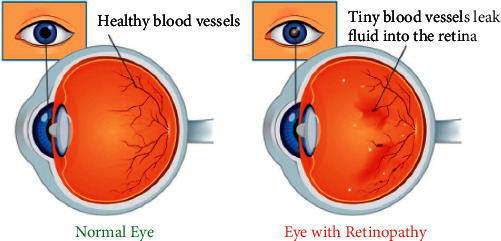
Diabetes retinopathy.

Diabetes has a dangerous effect on the eyes' small blood vessels. As a result, it may cause problems such as glaucoma (a condition in which fluid builds up in the eyes), cataracts (clouding of the lens of the eyes), and diabetes retinopathy (a condition in which the retina at the back of the eye is damaged). Diabetes can cause visual loss or blur over time. As a result, regular eye examinations by a qualified ophthalmologist are required. Early detection can help to avoid serious consequences. According to polls, early identification can prevent blindness in patients with diabetes by 90 percent.  Figure 3 shows the normal eye and eye with retinopathy.

### 1.3. Gestational Diabetes Mellitus (GDM)

GDM is a serious and underappreciated threat to the health of mothers and children. High blood pressure, large birth weight babies, and obstructed labour are common pregnancy problems for women with GDM. Within five to ten years following delivery, around half of women with a history of GDM acquire type-2 diabetes. The prevalence of high blood glucose during pregnancy rises dramatically with age, peaking in women over 45. However, diabetes mellitus, most usually type 2, is discovered in roughly 5–10% of women with GDM after pregnancy. GDM is completely curable, but it necessitates close medical monitoring during pregnancy.

## 2. Related Work

The utilization of ML approaches to improve the precision of DM risk prediction has been studied in a variety of ways [6]. The authors [7] have done a comparison of the accuracy of numerous algorithms on the dataset of diabetes. They discovered that the J48 algorithm has 73.82% accuracy before data preprocessing, which is superior to others. Following preprocessing, both KNN and RF showed better performance. In this work [8], J48, KNN, and LR were compared on the diabetes dataset in this work. J48 was found to be the most accurate, with a classification accuracy of 78.27%. Based on its accuracy of 80%, this work [9] created web-based software for DM detection. In this work, the authors compared prediction techniques such as decision trees (DT), neural networks, Naive Bayes (NB), LR, and RF, and ensemble techniques. The authors found that RF performed the best in terms of accuracy and ROC score of 75.558% and 0.912, respectively.

The authors in this [10] looked at different ML algorithms on a medical dataset. They compared different performance indicators. In this research, ML and data mining approaches used in DM research were identified and reviewed in a systematic manner. Diabetes mellitus is quickly becoming a very important and pressing worldwide health issue of the twenty-first century. At present, significant research has been conducted in practically every facet of DM research. When compared to other ML algorithms, authors [11] employed C4.5 with 78.25% accuracy. According to them, J48 is famous for its correctness.

The authors of this article [12] present an overview of how DM is linked to cardiovascular autonomic neuropathy that impairs HRV. As a result, diabetes was discovered using HRV spectral analysis. Using DWT features retrieved from HRV signals, the authors demonstrated an automated DM detection technique. Using the DT classifier, they were able to get sensitivity, accuracy, and specificity of 92%, 92.59%, and 91.46%, respectively, using their proposed method. In their diabetes forecasting experiments, the authors looked into NB, ANN, as well as SVM classifiers [7]. They conducted a research study that was weighted. The majority vote method was used in this investigation. The findings show that a combination of classifiers outperformed any individual classifier.

The authors of this study [13] examined different ML algorithms on the Pima Indian dataset. Feature selection was applied to the dataset. According to their findings, attribute selection improved the performance of diabetes prediction. This study's retrospective cross-sectional methodology prevents the authors from establishing a cause-and-effect link. This strategy may simply be utilized in studies to find the optimal type-2 diabetes phenotype or prediction in different nations. However, because the study group was limited to Korean women and men, the findings could not be extended to other populations. There can be disparities in socioeconomic class, race, gender, and nationality within a single patient cohort.

Using the J48 DT, the authors [6] emphasized the application of Adaboost ensemble methods for diagnosing the disease. According to the findings of the trial, Adaboost ensemble approaches outperform J48 and even bagging. In this study, the scientists employed NB, J48, and RF for the prediction of DM. In terms of accuracy, they discovered that RF outperforms naive Bayes and J48 techniques. According to the authors [14], the accuracy achieved by implementing PCA was not good, but the outcome of using all features along with MRMR was superior. The results showed that by using fasting glucose made the performance better, particularly in the Luzhou dataset. This implies that, while fasting glucose is the most significant predictor, it cannot produce the best results on its own, so multiple indices were required to effectively predict. Furthermore, they discovered that there is little difference between RT, DT, and neural network results when comparing the three classifications; however, RFs are definitely better compared to the other three classifiers. The highest performance for the Luzhou dataset was 80.84%, while that of the Pima Indians dataset was 77.21%, indicating that ML can be used to predict diabetes, but selecting appropriate features, classifiers, and data mining methods is crucial. According to the results, the Adaboost ensemble technique outperforms bagging and the standalone J48 DT in this study [15].

Several ML algorithms for diabetes mellitus prediction were utilized in the studies listed above, and they have been evaluated for getting good results. In this article, the ML techniques DT, RF, LR, SVM, and KNN classifiers are utilized as base learners, and their performance is compared to that of the ensemble classifier using various performance measures, namely, accuracy, precision, sensitivity, and f1-measure in the prediction of diabetes.

## 3. Methodology

**Figure 4:**
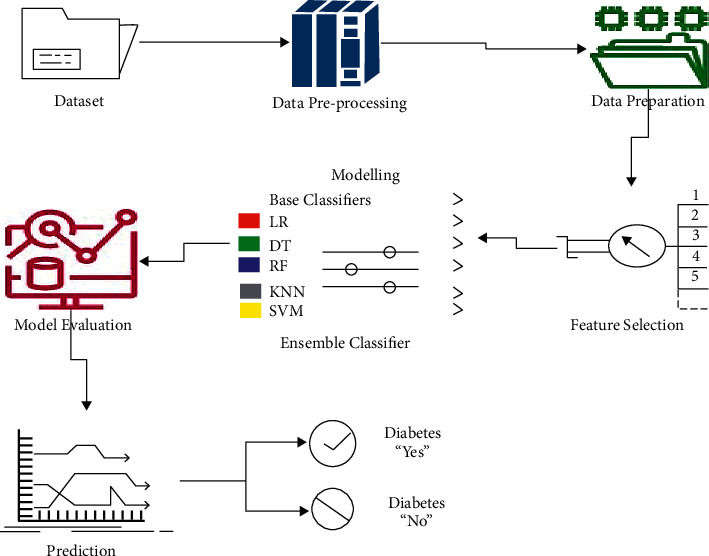
Different phases of the prediction process.

The likelihood of survival and the likelihood of diabetes recurrence are highly dependent on medical treatment and the accuracy of the diagnosis. The various phases of the prediction process of diabetes are depicted in Figure 4.

The dataset taken from Kaggle consists of 693 diabetes-affected people's records. The different symptoms along with their descriptions are shown in Table 1. Arbitrarily extracted information was used in this investigation, with a ratio of 75 : 25 split between training and testing data. The model was trained to utilize training sets (520 records), and its effectiveness was tested via test data. The dataset consists of 16 features or attributes whose values will determine whether the person is likely to be affected by diabetes or not. The target or output variable has a value of 1 for the presence of diabetes and 0 for its absence.

### 3.1. Logistic Regression (LR)

This approach has been modified for binary classification problems. The fundamental goal of LR is to figure out what the coefficients are worth. The LR reduces the value to a number between 0 and 1. The LR model chooses a probability of 0 or 1 for the provided data instance of the class to predict. This method can be used to solve problems where we have various reasons to forecast. Equation (1) is the definition of the LR standard function:

(1) $$\begin{array}{c} h\theta \left(X\right)=\frac{1}{1+e^{-\left(\beta_{0}+\beta_{1}X\right)}}. \end{array}$$

The constants are represented in *β*_1_ and *β*_0_ by the data label *X*.

### 3.2. K-Nearest Neighbor (KNN)

KNN is also a supervised ML algorithm [11]. It is mostly used to solve categorization challenges. The object is categorized using K-neighbors. Before running the procedure, the positive number K must be defined. Euclidean distance is frequently used to determine various object measurements [16]. The Euclidean distance and Manhattan distance are calculated using the following equations:

(2) $$\begin{array}{c} \text{Euclidean}=\sqrt{\sum_{i=1}^{k}{\left(a_{i}-b_{i}\right)}^{2}}, \end{array}$$

(3) $$\begin{array}{c} \text{Manhattan}=\sum_{i=1}^{k}|a_{i}-b_{i}. \end{array}$$

From the above equations, Euclidean as well as Manhattan of KNN is computed with *a* and *b* data upto *I* variables.

### 3.3. Support Vector Machine (SVM)

The SVM algorithm [17] is a supervised ML approach. For a small data set with a few outliers, this model is ideal. The goal is to determine the hyperplane that will be used to split the data points. The hyperplane will be used to divide two spaces into different domains. Similar forms of data will be found in such a domain. The support vector machine's decision state is represented by the following equation:

(4) $$\begin{array}{c} \left|\left|Y\right|\right|=\sqrt{y_{1}^{2}+y_{1}^{2}+\cdots +y_{n}^{2}}. \end{array}$$

### 3.4. Decision Tree (DT)

**Figure 5:**
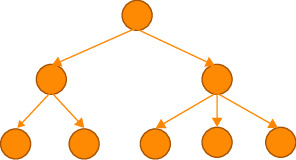
A single DT.

DT is one technique to show an algorithm that is made up of completely conditional control statements. The DTs are a collection of well-known supervised classification algorithms. They do well on classification problems; the decisional path is straightforward to interpret, and the algorithm for building (training) them is quick and very simple. A single DT is depicted in Figure 5.

### 3.5. Random Forest (RF)

**Figure 6:**
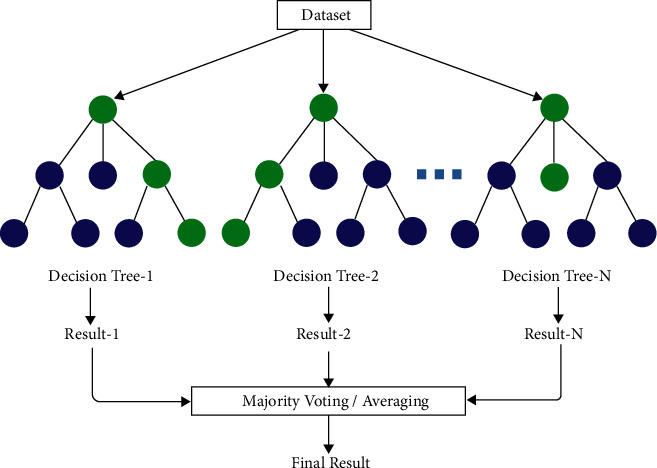
RF example.

RF is an ML technique that is part of the supervised ML model. The RF classifier is made up of numerous decision trees representing various subjects [18]. It takes the average of each tree's subset to improve predictive accuracy. RF, rather than depending on a single decision tree, uses the majority vote prediction technique from all the trees and then predicts the result [19]. Every node in the decision tree answers a query about the situation. RF example is depicted in Figure 6.

### 3.6. Proposed Ensemble Learning (EL) Technique

**Figure 7:**
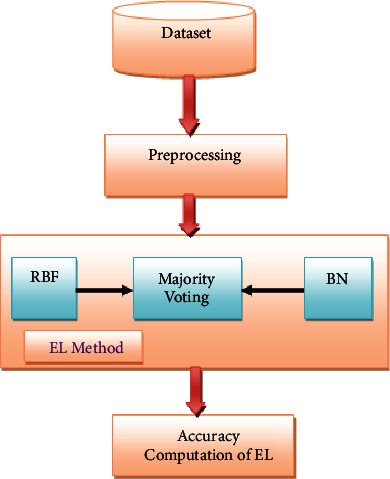
Proposed process diagram.

For the categorization of diabetes mellitus, an ensemble classifier based on Bayesian Networks and Radial Basis Functions is proposed in this article [20]. Ensemble classifiers are modelled using Bayesian networks (BNs) as well as Radial Basis Function (RBF). Two individual classifiers and the suggested ensemble classifier will be discussed in the subsections.  Figure 7 depicts the suggested approach's process flow diagram.

#### 3.6.1. Bayesian Network (BN)

Traditional statistical models do not allow for the incorporation of past knowledge into the model. The probabilistic associations between items or objects are represented using a BN [21]. BNs are directed acyclic graphs with nodes indicating variables and missing edges representing conditional independence between variables. The probability function is assigned to each node. Bayesian networks are used to represent knowledge in an area that is uncertain. Statistics, probability theory, graph theory, and computer science are all combined in Bayesian Networks [22].

BN depicts a joint probability distribution over a set of random variables “*X.*” The BN is explained as *Y*=〈*G*, *θ*〉 where *G* is *DA* *G*. It contains the nodes *N*_1,_*N*_2_,…,*N*_*n*_, and edges depict dependencies among the variables. Joint distribution for a BN is defined as shown in equation (5).

*P* (nodes|parents (node) ) for all nodes

(5) $$\begin{array}{c} P\left(X_{1},\ldots \ldots ,X_{n}\right)=\prod_{i=1}^{n}P\left(X_{i}|X_{1},\ldots \ldots ,X_{i-1}\right)\\ =\prod_{i=1}^{n}P\left(X_{i}|\text{Parents}\left(X_{1i}\right)\right). \end{array}$$

When huge networks are used, this combined distribution will reduce computation. The use of a Bayesian network is motivated by the fact that they record high accuracy for complicated and uncertain domains.

#### 3.6.2. Radial Basis Function (RBF)

The supervised algorithm RBF is derived from function approximation. RBFs have three layers: an I/P layer, an O/P layer, and a hidden layer [23]. Each hidden unit in the input space has its own receptive field. These units are known as radial centers, and they are denoted by the letters *V*_1_, *V*_2_,…, *V*_*n*_. The transformation from hidden units to output is linear but not from the input layer to the hidden unit. RBF will create a local map, as a result of which it will learn quickly. RBF employs two-step learning, requiring the acquisition of both weight and centers. By calculating the input's similarity to instances from the training set, RBF achieves supervised learning. Each neuron computes the distance between the input and its prototype to classify a fresh sample. The Gaussian equation is shown as follows:

(6) $$\begin{array}{c} f\left(x\right)=\frac{1}{\sigma \sqrt{2\pi }}e^{-\left({\left(x-u\right)}^{2}/2\sigma^{2}\right)}, \end{array}$$

where *x* is the input, *u* is the mean, and *σσ* is the standard deviation.

The neuron activation function of RBF is defined as shown in the following equation:

(7) $$\begin{array}{c} \emptyset \left(x\right)=e^{-\beta |\left|x-\mu \right|{|}^{2}}. \end{array}$$

The RBF is motivated by the fact that it learns faster than a simple feed-forward network because of the following advantages: training is quick, MLP output nodes implement linear summation, they are good at interpolation, and radial basis functions are implemented by hidden nodes.

#### 3.6.3. Ensemble Learning (EL) Method

Ensemble learning is an effective machine learning technique for improving model performance. The model's prediction capability is improved by combining the varied sets of learners (Base Learners). The importance of selecting the appropriate ensemble for diabetes prediction cannot be overstated.

RBF and Bayesian Network have been employed as base learners in the proposed methodology, which was then integrated with the EL method. Majority voting can be represented as shown in the following equation:

(8) $$\begin{array}{c} \sum_{t=1}^{T}d_{t,J}=\frac{C}{\max_{j=1}}\sum_{t=1}^{T}d_{t,j}, \end{array}$$

where T is the dataset and C is the class label.

## 4. Results and Discussion

**Figure 8:**
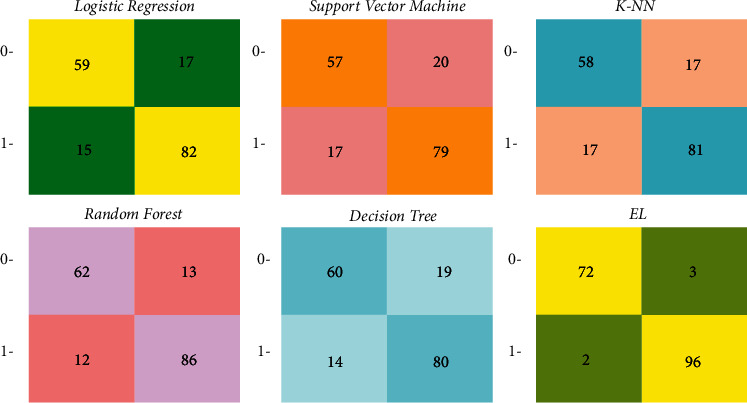
Confusion matrix.

A confusion matrix is a matrix that comprises data on actual and expected classifications and is used to assess the algorithm's performance using the matrix data. We get both correct and incorrect predictions from each classifier. False Positive and False Negative predictions are two types of incorrect predictions. When we forecast that something will happen/occur but it does not, we call it a False Positive also called as type I error (a valid null hypothesis is rejected). For example, we predicted that diabetes would develop, but it did not. When we forecast that something will not happen/occur, but it does, we call it a False Negative also called as type II error (the failure to reject a false null hypothesis). For example, we might anticipate that there will be no diabetes, but diabetes does exist. Type I errors are typically thought to be less dangerous than type II faults. In disciplines, such as medicine, both errors may appear to be fatal. The confusion matrix of all the ML algorithms is depicted in Figure 8.

The entire aforementioned models have been evaluated against precision, *F*1-measure, and recall, and the values obtained are depicted in Table 2.

The precision is computed using the following equation:

(9) $$\begin{array}{c} \text{precision}=\frac{\text{TPs}}{\text{TPs}+\text{FPs}}. \end{array}$$

The precision of LR, KNN, RF, DT, SVM, and EL classifiers is 82.82%, 82.65%, 86.86%, 80.80%, 79.79%, and 96.96%, respectively.

Recall is the proportion of TPs out of TPs and FNs. The recall is computed using the following equation:

(10) $$\begin{array}{c} \text{recall}=\frac{\text{TPs}}{\text{TPs}+\text{FNs}}. \end{array}$$

The recall of LR, KNN, RF, DT, SVM, and EL classifiers is 84.53%, 82.65%, 87.75%, 85.10%, 82.29%, and 97.96%, respectively.

The *F*1-score which is also called as harmonic mean that considers both precision as well as recall is computed using the following equation:

(11) $$\begin{array}{c} F1-\text{score}=2*\frac{\text{precision}*\text{recall}}{\text{precision}+\text{recall}}. \end{array}$$

**Figure 9:**
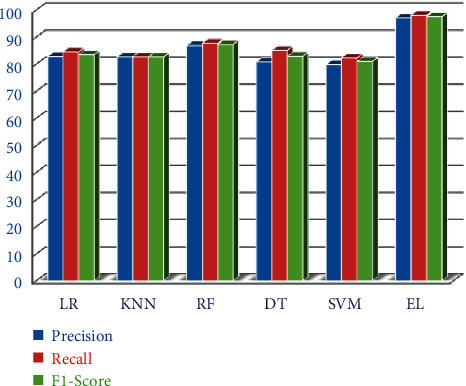
Performance of various ML techniques.

The *F*1-score of LR, KNN, RF, DT, SVM, and EL classifiers is 83.51%, 82.65%, 87.30%, 82.89%, 81.02%, and 97.46%, respectively. The recall, precision, and *F*1-scores of all the techniques are shown in Figure 9.

The number of correctly classified data instances divided by the total number of data instances is known as accuracy. The accuracy is computed using the following equation:

(12) $$\begin{array}{c} \text{accuracy}=\frac{\text{TNs}+\text{TPs}}{\text{TNs}+\text{TPs}+\text{FPs}+\text{FNs}}. \end{array}$$

**Figure 10:**
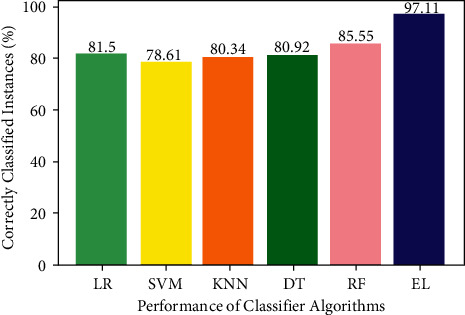
Accuracy of algorithms.

The accuracy of the different ML algorithms is also computed. The accuracy of LR, SVM, KNN, DT, RF, and EL classifiers is 81.50%, 78.61%, 80.34%, 80.92%, 85.55%, and 97.11%, respectively. The EL method has outperformed with an accuracy of 97.11% in comparison to other ML algorithms.  Figure 10 shows the accuracy performance of the aforementioned ML algorithms.

**Figure 11:**
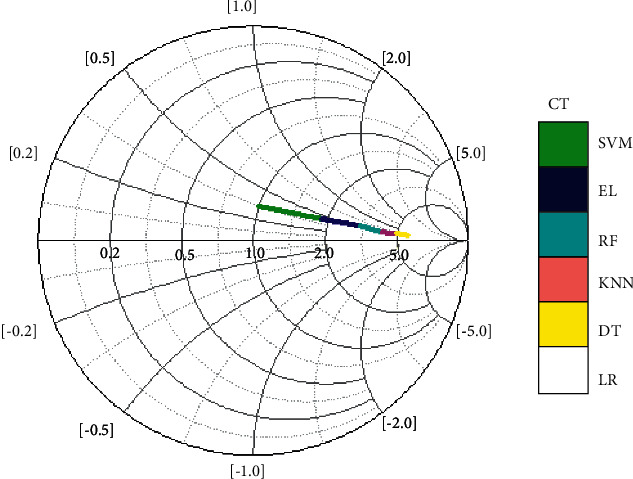
Computation analyses of ML techniques.

Figure 11 illustrates the processing time required for several classification techniques. In comparison to other methods, EL techniques take longer to process data due to the blending process of base learners. CG (conjugate gradient) optimization was utilized for primary LR with compliance. *L*2 regularization and Alpha = 0.0001 were being used by SVM to get the best learning rate. Using Gini impurity, a DT was divided into smaller trees, which consumed optimal time for processing. It was enlarged until all leaves were pure or included fewer than two nodes, whichever came first.

## 5. Conclusion

If identified early enough, diabetes has the potential to save thousands of lives. The main purpose of this article is to summarize all of the recent and ongoing investigations using machine learning algorithms for diabetic illness prediction. In addition, this study provides all of the relevant and required information for researchers who are new to deep learning and wish to examine ML methods. In conclusion, EL demonstrates its power in terms of efficacy and efficiency, as well as precision, recall, accuracy, and *F*1-score. The blended EL method provides the best accuracy of 97.11% compared to other models. More study is needed in this field to increase the performance of categorization systems so that they can forecast a larger number of variables. The goal is to figure out how to parameterize our categorization algorithms in order to attain high accuracy. A number of datasets are continuously being investigated to explore how ML techniques might be used to better predict diabetes.

## Figures and Tables

**Algorithm 1:**
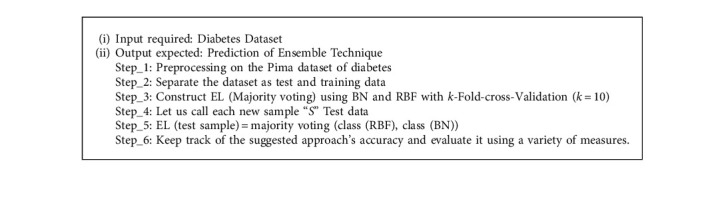
Stages in the proposed ensemble learning (EL) model.

**Table 1:** Feature descriptions.

Attribute/feature	Description
Age	Between 20 years and 90 years
Gender	1 denotes male, 0 denotes female
Polyuria	1 denotes male, 0 denotes female
Polydipsia	1 denotes male, 0 denotes female
Sudden weight loss	1 denotes male, 0 denotes female
Weakness	1 denotes male, 0 denotes female
Polyphagia	1 denotes male, 0 denotes female
Genital thrush	1 denotes male, 0 denotes female
Visual blurring	1 denotes male, 0 denotes female
Itching	1 denotes male, 0 denotes female
Irritability	1 denotes male, 0 denotes female
Delayed healing	1 denotes male, 0 denotes female
Partial paresis	1 denotes male, 0 denotes female
Muscle stiffness	1 denotes male, 0 denotes female
Alopecia	1 denotes male, 0 denotes female
Obesity	1 denotes male, 0 denotes female
Class	1 denotes positive, 0 denotes negative

**Table 2:** Performance metrics of different ML techniques in %.

ML techniques	Precision	Recall	*F*1-score
LR	82.82	84.53	83.51
KNN	82.65	82.65	82.65
RF	86.86	87.75	87.30
DT	80.80	85.10	82.89
SVM	79.79	82.29	81.02
**EL (BN** **+** **RBF)**	**96.96**	**97.96**	**97.46**

## Data Availability

The Irvine ML Repository data used to support the findings of this study are available at https://archive.ics.uci.edu/ml/datasets/.

## References

[1] Rish I. (2001). An empirical study of the naive Bayes classifier. *IJCAI*.

[2] Mahesh T. R., Vinoth Kumar V., Vivek V. (2022). Early predictive model for breast cancer classification using blended ensemble learning. *International Journal of System Assurance Engineering and Managament*.

[3] Fayyad U., Piatetsky-Shapiro G. P., Smyth P. (1996). The KDD process for extracting useful knowledge from volumes of data. *Communications of the ACM*.

[4] Fayyad U., Piatetsky-Shapiro G., Smyth P. (1996). From data mining to knowledge discovery in databases. *AI Magazine*.

[5] Mcculloch W. S., Pitts W. (1990). A logical calculus of the ideas immanent in nervous activity. *Bulletin of Mathematical Biology*.

[6] Abadi M., Barham P., Chen J. (2016). Tensorflow:A system for large-scale machine learning. *OSDI*.

[7] Acharya U. R., Vidya K. S., Ghista D. N., Lim W. J. E., Moli-nari F., Sankaranarayanan M. (2015). Computer-aided diagnosis of di-abetic subjects by heart rate variability signals using discretewavelet transform method. *Knowledge-Based Systems*.

[8] Chen X. X., Tang H., Li W. C., Wu H., Chen W., Ding H., Lin H (2016). Identification of bacterial cell wall lyases via pseudo amino acid composition. *BioMed Research International*.

[9] Habibi S., Ahmadi M., Alizadeh S. (2015). Type 2 diabetes mellitus screening and risk factors using decision tree: results of data mining. *Global Journal of Health Science*.

[10] Kavakiotis I., Tsave O., Salifoglou A., Maglaveras N., Vlahavas I., Chouvarda I. (2017). Machine learning and data mining methods in diabetes research. *Computational and Structural Biotechnology Journal*.

[11] Lee B. J., Kim J. Y. (2016). Identification of type 2 diabetes risk factors using phenotypes consisting of anthropometry and triglycerides based on machine learning. *IEEE Journal of Biomedical and Health Informatics*.

[12] Gowramma G. S., Mahesh T. R., Gowda G. (2017). An automatic system for IVF data classification by utilizing multilayer perceptron algorithm. *ICCTEST*.

[13] Sharma K., Mahesh T. R, Bhuvana J (2021). Big data technology for developing learning resources. *Journal of Physics: Conference Series*.

[14] Deshmukh A. A., Dubal M., Mahesh T. R, Chauhan C. R. (2012). Data security analysis and security extension for smart cards using java card. *International Journal of Advanced Information Technology (IJAIT)*.

[15] Mahesh T. R., Prabhanjan S., Vinayababu M. (2010). Noise reduction by using fuzzy image filtering. *Journal of Theoretical and Applied Information Technology*.

[16] Shashikala H. K., Mahesh T. R., Vivek V., Sindhu M. G., Baig C., Baig T. Z. Early detection of spondylosis using point-based image processing techniques.

[17] Dubai M. J., Ghosh T. R., Ghosh P. A. Design of new security algorithm: using hybrid Cryptography architecture.

[18] Perveen S., Shahbaz M., Guergachi A., Keshavjee K. (2016). Performance analysis of data mining classification techniques to predict diabetes. *Procedia Computer Science*.

[19] Mahesh S., Mahesh T. R., Vinayababu M. (2010). Using data mining techniques for detecting terror-related activities on the web. *Journal of Theoretical and Applied Information Technology*.

[20] Ghosh P., Mahesh T. R. A privacy preserving mutual authentication protocol for RFID based automated toll collection system.

[21] Shukla P. K., Kaur Sandhu J., Ahirwar A., Ghai D., Maheshwary P., Shukla P. K. (2021). Multiobjective genetic algorithm and convolutional neural network based COVID- 19 identification in chest X-ray images. *Mathematical Problems in Engineering*.

[22] Pinaki G., Mahesh T. R. (2015). Smart city: concept and challenges. *International Journal on Advances in Engineering Technology and Science*.

[23] Mahesh T. R., Dhilip Kumar V., Vinoth Kumar V. (2022). AdaBoost ensemble methods using K-fold cross validation for survivability with the early detection of heart disease. *Computational Intelligence and Neuroscience*.

